# Evaluation of the Effects of Multifocal Intraocular Lens Oculentis LENTIS Mplus LS-313 MF30 on Visual Performance in Patients Affected by Bilateral Cataract and Treated with Phacoemulsification

**DOI:** 10.1155/2022/1315480

**Published:** 2022-08-30

**Authors:** R. Nuzzi, F. Tripoli, A. Ghilardi

**Affiliations:** Department of Ophthalmology, Department of Surgical Sciences, University of Turin, Via Cherasco 23, Turin, Italy

## Abstract

**Background:**

The purpose of this study was to evaluate the visual results and patients' satisfaction with surgical treatment of phacoemulsification and implantation of the innovative intraocular multifocal lens (MFIOL) Oculentis LENTIS Mplus MF30 in patients with bilateral cataracts.

**Materials and Methods:**

A single-center prospective observational study was conducted on a total of 20 patients with bilateral cataracts. We evaluated the monocular UCVA and BCVA at 1 day, 7 days, 1 month, 3 months, and 6 months at different distances after phacoemulsification and MFIOL implantation and the binocular UCVA at the same distances. We also assessed the frequency of visual disturbances, overall visual satisfaction, spectacles dependence, and ease of performing different daily activities.

**Results:**

The MFIOL Mplus MF30 was able to significantly improve the monocular UCVA and BCVA at all working distances. Overall visual satisfaction was above 9/10 in all postoperative observation intervals. The degree of independence from spectacles at all distances was 100%. The frequency of adverse visual phenomena was minimal.

**Conclusions:**

In accordance with the literature, the Oculentis LENTIS Mplus MF30 has proved to be a valid therapeutic alternative for visual rehabilitation after phacoemulsification of the cataract in patients also wishing to treat presbyopia, at the cost of very few visual adverse effects. *Trials Registration*. This trial is registered with ISRCTN20862627.

## 1. Background

To the present day, the gold standard in cataract treatment is the surgical procedure known as phacoemulsification of the lens, followed by the implant of an intraocular lens to replace the native one.

In the past 50 years, cataract surgery's success would have been incomplete and less remarkable without the constant development and improvement of IOLs technology, which can anatomically and functionally replace crystalline [1–3].

Monofocal IOLs are the most frequently used type of lenses, and they are implanted after surgery to restore distance vision or near distance vision and lens transparency. Their critical flaw is that patients will invariably have to wear spectacles to perform tasks at an intermediate distance and close-up distance [4].

To restore patients' ability to see at close distances without the need for spectacles, multifocal IOLs (MFIOLs) have been under constant development since 1980 [5]. This type of IOLs has a peculiar optical design that creates multiple images of the same object and makes light rays focalize on the retina's different points [4, 6, 7]. Through a process of neuroadaptation and habit, patients can select the image that falls closer to the retina and provides the object's clearest perception, neglecting other images that could give confused or blurred vision. The ability of visual brain centers to suppress other images allows the patient to avoid monocular diplopia [6, 8–10].

MFIOLs are mainly distinguished by their optical design [11]:

  Refractive MFIOLs have rotationally symmetrical designs, and their surface has two or more concentric rings with different curvature radii and optical power, creating bifocal or multifocal vision. Additionally, refractive MFIOLs may determine adverse effects such as halos or glares caused by irregularities in the lens areas where different optical surfaces are in contact.
  Diffractive MFIOLs have multiple concentric rings on their surfaces with diffractive microstructures separated by 2 µm high “steps.” These microstructures create a diffractive wave pattern that can focalize light rays on two or more foci. Their main flaw is related to energy loss by light rays while passing through diffractive surfaces, an inconvenience that can reduce contrast sensibility in some patients. Furthermore, diffractive lenses can more frequently cause halos or glares because of their multiple diffractive surfaces.

Spectacle independence is a vastly growing expectation among patients that undergo a cataract surgical correction. They frequently need and ask for good visual quality and acuity while performing various tasks that require them to shift between different visual distances [4, 12, 13].

MFIOLs have proved to be one of the most effective and safe treatments for middle-aged (>50 years old) patients affected by cataracts and presbyopia. While MFIOLs can restore lens transparency, they also allow patients to achieve optimal visual quality and acuity at all visual distances. The degree of satisfaction and quality of vision is better if MFIOLs are proposed and implanted in wisely selected patients who are strongly motivated to achieve spectacle independence, albeit at the cost of some minor adverse effects [6, 8, 10, 14, 15].

The aim of this observational, prospective, single-site study is to evaluate visual performance and quality outcomes at different working distances—far (4 m), intermediate (60 cm), and near (33 cm)—of the innovative Oculentis LENTIS® Mplus LS-313 MF30, a rationally asymmetrical MFIOL specifically designed to reduce the typical adverse effect of other multifocal IOLs [16, 17].

While the literature has already considered and evaluated MFIOLs and various Mplus-type MFIOLs from different points of view, this study is focused on the clinical evaluation of LS-313 MF30 implantation and its consequences on patients' visual acuity and quality, especially after bilateral implantation.

## 2. Materials and Methods

This study was conducted at the Ophthalmology Department of A.O.U. Città Della Scienza e Della Salute of Turin, Italy, between April 2018 and September 2020.

Our institutional Ethics Committee has been consulted; before starting the experimentation, formal approval was obtained. Informed consent was obtained from each recruited subject before the surgical treatment.

We recruited candidates for bilateral cataract extraction surgery. Each subject underwent complete ophthalmologic evaluation 90 days before the procedure [4] (as part of a standard preoperative practice), with:

  Collection of personal data and anamnesis
  Slit- lamp ocular examination of the anterior segment and fundus oculi
  Ocular applanation tonometry with Goldman tonometer
  Determination of refraction and UCVA (uncorrected visual acuity) and BCVA (best-corrected visual acuity) for both eyes at 4 m, 60 cm, and 33 cm with ETDRS and Jaeger tables
  Keratometry with Javal's ophthalmometer
  Optical eye biometry (Topcon Aladdin®) and contact eye biometry (we observed no differences between these two procedures; *p* > 0.1)
  Corneal surface topography with Topcon Aladdin®
  Photopic and mesopic pupillometry with Topcon Aladdin®

Inclusion criteria were as follows:

  Bilateral cataract
  Age >50 years
  Regular corneal astigmatism, <1.00 *D*
 
*K* angle ≤1 mm

We excluded patients with opacities of optic media other than cataract, age-related macular degeneration, previous history of ocular surgery, irregular corneal astigmatism, amblyopia, concurrent neurologic or neuromuscular diseases (cerebral ictus, myasthenia gravis), uncontrolled open/close-angle glaucoma, severe ocular complications related to diabetes (retinopathy, macular edema, vitreal hemorrhage), and patients with operative or postoperative complications. Patients with a pupil diameter of ≤5.2 mm in mesopic lighting conditions were also excluded from the study.

We recruited 20 patients for a total of 40 eyes.

Each patient received a preoperative prophylactic treatment with moxifloxacin eye drops (5 mg/mL), one drop three times each day for three days before the surgical procedure. Afterwards, each patient underwent bilateral cataract extraction surgery. Each eye was treated at a different session, 30 days apart.

All surgical procedures were performed by the same surgical équipe and consisted in the following:

  Preoperative application of local mydriatic agents (Mydriasert) 1 h before surgery.
  Preoperative application of local anesthetics (Benoxinate, 0.4%)
  Sterile field preparation and disinfection with povidone-iodine for 3 minutes
  Corneal tunnel incision of 2.2 mm and paracentesis followed by introducing viscoelastic fluid (Viscoat and Visthesia) in the anterior chamber
  Execution of continuous circular capsulorhexis
  Nucleus hydrodissection
  Cataract extraction with phacoemulsification technique
  MFIOL LENTIS Mplus LS-313 MF30 implantation in the capsular bag
  Removal of viscoelastic fluid in front of and behind the lens
  Hydrosuture
  Disinfection and medication

None of the recruited patients had significant astigmatism (see inclusion criteria). After ocular biometry, the choice of IOL was made based on the SRK/T®, Holladay I, and Hoffer *Q* formulas. Although the lens manufacturer suggests selecting the IOL that guarantees the least myopic residual [16], we opted for the MFIOL that would determine the least hypermetropic shift for all patients, bilaterally. This choice was dictated by clinical experience in previous research and allowed us to obtain good results, as explained below [4].

After surgery, patients were kept under observation for a few hours and later discharged according to standard protocol. They were subsequently asked to show up for follow-up ophthalmologic visits after 1 day (t1), 7 days (t2), 1 month (t3), 3 months (t4), and 6 months (t6). On all those occasions, we performed slit-lamp eye examination, ocular tonometry, refraction determination, and monocular UCVA and BCVA evaluation for each eye at 4 m, 60 cm, and 33 cm with ETDRS tables and Jaeger tables. We evaluated the binocular UCVA at 4 m, 60 cm, and 33 cm in the last three visits. In the same time intervals of 1 month, 3 months, and 6 months, we also assessed patients' satisfaction, personal spectacle independence, the incidence of adverse visual effects, and the treatment effect on different daily activities with specific questionnaires.

We confronted the preoperative monocular and binocular visual acuity with the postoperative visual acuity measured in the Student's *T*-test's follow-up intervals.

We also assessed all single-eye observations' overall visual acuity with the Student's *T*-test to determine whether additional lenses could achieve a better visual acuity. We inferred statistical significance with a *p*-value <0.05.

All procedures in this study were performed in conformity with ethical principles presented in the Helsinki Declaration.

## 3. Results

Twenty patients for a total of 40 eyes were treated with phacoemulsification and bilateral MFIOL implantation. Within the time required to complete the follow-up (6 months), no patient failed to show up at follow-up visits, and no patient experienced complications such that they should be excluded from the study.

### 3.1. Comparison between the UCVA at t0 (Preoperative) and the UCVA at t5 (6 months Postoperative) and between the BCVA at t0 and the BCVA at t5 at 4 m, 60 cm, and 33 cm

We recorded data regarding UCVA and BCVA for the right and left eyes at distances of 4 m, 60 cm, and 33 cm, and we performed a comparison between the UCVA at t0 and the postoperative UCVA at t5. Subsequently, we performed the same analysis between the BCVA at t0 and the postoperative BCVA at t5. A statistically significant benefit from the multifocal intraocular lens was observed in all cases. A detailed discussion of these results follows.

Before surgery (Table 1), patients had an average 4 m UCVA of 0.57 LogMAR for OD and 0.56 LogMAR for OS; BCVA was 0.35 LogMAR and 0.31 LogMAR for OD and OS, respectively. In no case, a statistically significant difference between the two eyes (OD vs. OS) was observed. At t5, we noted a statistically significant increase in UCVA at 4 m for both OD and OS: 0.57 versus 0.12 LogMAR (*p* < 0.001) and 0.56 versus 0.11 LogMAR (*p*=0.001), respectively. BCVA at 4 m was also significantly better at t5 for both eyes: 0.35 versus 0.07 LogMAR (*p* < 0.001) for OD and 0.31 versus 0.07 LogMAR (*p*=0.006) for OS.

The same data collection was performed for VA at intermediate distances (60 cm), as reported in Table 2. On average, before surgery, patients had a UCVA of 11.1 J in OD and 10.5 J in OS. BCVA was 7.1 and 6.8 J, respectively. No significant difference was observed between the two eyes. After surgery, UCVA improved for both eyes to 2.6 J in OD and 2.3 J in OS. We noted a significant difference between the UCVA values at t0 versus t5 (p < 0.001). Sixty  centimeter BCVA at t5 was also better than the preoperative BCVA: 7.1 J versus 2.0 J for OD (*p* = 0.001) and 6.8 J versus 2.0 J for OS (*p* = 0.002).

The MFIOL performance is further validated by the data referable to VA at a distance of 30 cm, reported in Table 3. We observed that patients had, on average, a t0-UCVA of 8.6 J and 8.1 J in OD and OS, respectively. BCVA mean values were 2.7 J for OD and 3.0 J for OS. At t5, UCVA increased for both OD and OS: 1.1 versus 8.6 J (*p* < 0.001) and 1.2 versus 8.1 J (*p* < 0.001), respectively. After surgery, t5-BCVA also improved significantly: 1.0 versus 2.7 J in OD (*p* = 0.035) and 1.0 versus 3.0 in OS (*p* = 0.026). Furthermore, we observed that at the end of the follow-up period (6 months), all patients enrolled in this study had achieved an average 30 cm UCVA of 1.1 J in OD and 1.2 J in OS, without significant differences between the two eyes, confirming the good performance of Mplus MF30.

### 3.2. Comparison between Mean Monocular UCVA and BCVA at 4 m, 60 cm, and 33 cm at t1–t5

We then merged all single observations regarding both OD and OS visual acuity. Therefore, we obtained 20 x 2 = 40 independent observations of monocular visual acuity at the previously specified visual distances at different time intervals.

**Figure 1:**
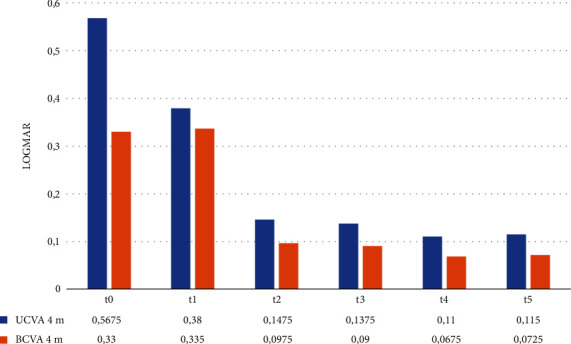
Mean monocular 4 m UCVA and BCVA at t0–t5.

Figure 1 shows the progression of UCVA and BCVA at a distance of 4 m during the follow-up intervals. At t0, we observed a sharp difference between the average UCVA and BCVA. At t1, the difference between the two columns is reduced. However, we noted how transiently the t1-UCVA is reduced compared to t0-BCVA, while t1-BCVA remains virtually unchanged compared to the t0-BCVA. This phenomenon is easily explained by the presence of residual inflammatory reaction 24 hours after surgery. In fact, at the subsequent observation interval t2, a net increase in both UCVA and BCVA is observed, which remains almost constant in the subsequent follow-up intervals t3–t4–t5. We observed that on average, all patients had an excellent UCVA and BCVA already at t2, 1 week after surgery.

**Figure 2:**
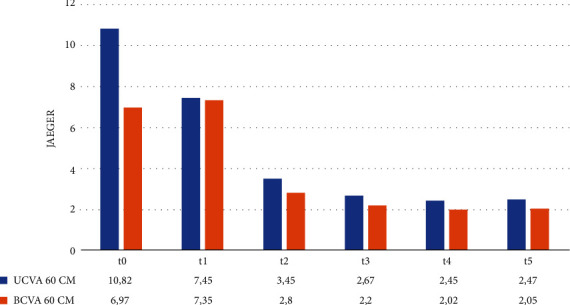
Mean monocular 60 cm UCVA and BCVA at t0–t5.

**Figure 3:**
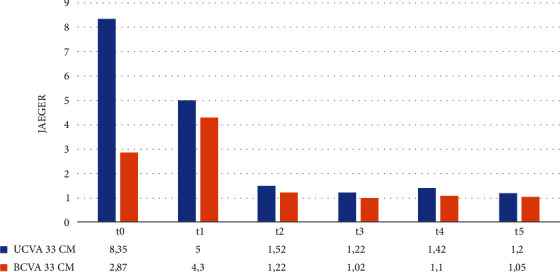
Mean monocular 33 cm UCVA and BCVA at t0–t5.

The same progression was observed both for 60 cm and for 33 cm, as shown in Figures 2 and 3.

### 3.3. Comparison between Mean Binocular UCVA Values in t0–t3, t3–t4, and t4–t5

We compared the mean binocular UCVA values in the time intervals t0–t3, t3–t4, and t4–t5 at distances of 4 m, 60 cm, and 33 cm. We observed a statistically significant improvement in mean binocular UCVA in the interval t0–t3 at all distances and that UCVA reached optimal levels already at t3 without additional lenses to further improve VA. Over the subsequent time intervals, the mean binocular UCVA remained constant with no statistically significant variability.

**Figure 4:**
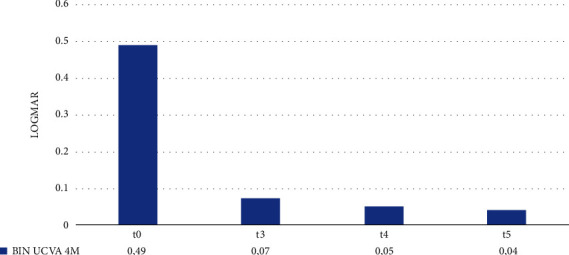
Binocular 4 m UCVA at t0, t3, t4, and t5.

Figure 4 shows the average values of binocular UCVA at a distance of 4 m at t0, t3, t4, and t5. The average binocular UCVA at t0 is 0.49 LogMAR. It significantly improved at t3, reaching the mean value of 0.07 LogMAR, and it remained stable throughout the follow-up period.

As reported in Table 4, we observed a statistically significant improvement in mean binocular UCVA at 4 m in the interval t0–t3. On the contrary, the difference in mean binocular UCVA in the t3–t4 and t4–t5 intervals was not statistically significant (*p*-value = 0.214 and 0.163, respectively).

Remarkably, 1 month after phacoemulsification surgery and subsequent Oculentis MF30 implantation in the second eye, patients reached, on average, a binocular UCVA of 0.0 LogMAR at a distance of 4 m with no need for additional lenses.

**Figure 5:**
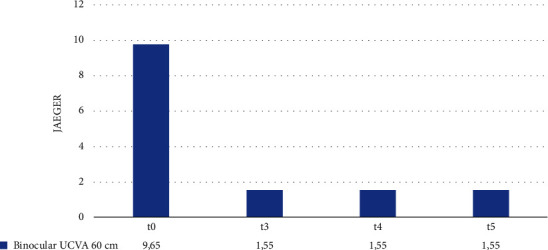
Binocular 60 cm UCVA at t0, t3, t4, and t5.

We performed the same statistical analysis for the mean binocular UCVA at a distance of 60 cm.  Figure 5 shows the average values of binocular UCVA at a distance of 60 cm at t0, t3, t4, and t5. Compared to the preoperative, t3-UCVA improved significatively and remained constant throughout the follow-up.  Table 5 shows the mean difference values of the binocular UCVA at 60 cm in the intervals t0–t3, t3–t4, and t4–t5. We noted that the improvement in the mean binocular UCVA in the interval t0–t3 is statistically significant (*p*-value <0.001). On the contrary, the changes in the mean binocular UCVA in the intervals t3–t4 and t4–t5 did not prove to be statistically significant (*p*-value = 1 and 1, respectively). We could argue that patients reached an optimal VA already 1 month after surgery and did not need additional lenses to further improve VA.

**Figure 6:**
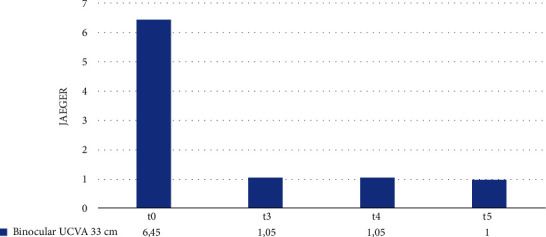
Binocular 33 cm UCVA at t0, t3, t4, and t5.

Ultimately, we assessed the mean binocular UCVA at a distance of 33 cm. As shown in Figure 6, at t0, the average binocular UCVA at 33 cm is 6.45 J. We observed a sharp increase at t3, with an average value of 1.05 J, which remained stable until the end of the follow-up period.

Table 6 compares mean binocular UCVA values at 33 cm in the intervals t0–t3, t3–t4, and t4–t5. The improvement in the mean binocular UCVA in the t0–t3 interval is statistically significant (*p*-value <0.001). In the interval t3–t4, no difference was found between the average values of binocular UCVA. In the interval t4–t5, a nonstatistically significant change in mean binocular UCVA is observed (*p*-value = 0.33).

MFIOLs are currently the best alternative to ensure that patients undergoing phacoemulsification have the highest degree of independence from the use of glasses. There is substantial agreement in the literature that they are effective in providing excellent visual recovery in distance vision although near vision and especially vision from intermediate distances are still critical [8, 18].

A multicenter study coordinated by Auffarth [16] in 2009 examined specifically the LENTIS® Mplus MF30, observing an average monocular BCVA in far vision at 6 months after surgery of 0.0 LogMAR as well as excellent 33 cm near vision. Similarly, a study by Venter [19] in 2013 reported a mean postoperative monocular UCVA of 0.05 LogMar at 4 m and 4 Jaeger at 30 cm. Our study seems to confirm these results.

In the vast landscape of multifocal IOLs, the literature seems to agree that refractive and diffractive IOLs perform equally well in terms of potential attainable visual acuity [20] although some work indicates a slight superiority of diffractive IOLs albeit at the cost of adverse visual effects [17, 21].

There is substantial evidence that MFIOLs better perform when they are bilaterally implanted [4, 22]. Berrow et al. [23], in 2014, assessed the visual performance of Mplus lenses by observing a mean postoperative monocular UCVA of 0.10 LogMAR and binocular of 0.02 LogMAR at 4 m and a mean postoperative monocular UCVA of 3 J and binocular of 2 J at 33 cm [23]. Our study comes to a similar conclusion: we recorded an average monocular UCVA at 4 m of 0.11 LogMAR 6 months after surgery and a mean binocular UCVA of 0.07 LogMAR 1 month after surgery, which remains constant until the end of the follow-up period.

## 4. Patient's Satisfaction

We evaluated patients' need to use additional lenses to achieve the desired VA, the incidence of adverse visual phenomena, and patients' ease in carrying out daily activities with specific questionnaires 1 month, 3 months, and 6 months after surgery in the second eye.

We assessed visual satisfaction on a scale from 0 (absolutely not satisfied) and 10 (fully satisfied). Patients expressed high satisfaction after surgery (>9), which remained constant. The difference between the visual satisfaction ratings between t3, t4, and t5 is not statistically significant, indicating that patients were immediately and continuously satisfied. The data relating to visual satisfaction are shown in Table 7.

The degree of spectacles independence was also assessed by asking patients how often they needed to use additional lenses to achieve the desired VA at different working distances. The evaluation scale ranged from a minimum of 0 (always need to wear lenses) to a maximum of 10 (no need to wear lenses). The results were highly satisfactory since all treated patients achieved almost complete independence from glasses at all distances. This result confirms the previous statistical analysis that showed that postoperative UCVA improved statistically significantly at all distances in binocular vision and that it was optimal already 1 month after surgery without the need to use additional lenses (Table 7).

We then analyzed the frequency of visual disturbances typical of MFIOLs [8, 24], such as luminous halos, glare, blurred vision, and double vision. As we previously stated, LENTIS® Mplus MF30 has an innovative design specifically intended to reduce such adverse effects. During the follow-up visits, we performed this survey by asking patients to express a quantitative judgment from 0 (absence of disturbances) to 5 (severe presence of disturbances), as reported in Table 8).

The most frequently experienced adverse visual symptom was the “glare.” We found no significant differences between the frequency of this disturbance between the examined time intervals, and on average, patients rated it as infrequent. Combining this observation with the previous data on visual satisfaction, it seems that this adverse effect did not significantly affect visual satisfaction.

The difficulty in night vision was very infrequent, and it remained constant at various intervals. The clear perception of colors [12] is a much-discussed topic in the literature about multifocal intraocular lenses, as MFIOLs could determine a reduction in contrast and color sensitivity. Nevertheless, none of the patients enrolled in this study reported having experimented with any of these adverse effects. Also, all patients reported that depth perception was not altered. Luminous halos, another frequent adverse photic phenomenon, represent the second most often adverse visual symptom found in our study, even though with a low incidence. Distorted near and far vision were rare, as well as was the perception of double images. These values remained the same throughout all the observation intervals. Blurred vision was the third most reported adverse effect although with low-frequency values. We observed that blurred far vision has been reduced between t3 and t4, although not singificantly (p>0.05); the blurred close-up vision has remained almost constant.

Table 9 shows how easily patients were able to perform various daily activities after surgery. The rating scale extends from 0 (total ease) to 5 (maximal difficulty). In general, we could observe that our patients generally reported ease in carrying out these daily activities. They reported having a high degree of ease in doing outdoor work, reading the time at a close distance on an alarm clock and a wall clock, caring for children, cooking, shopping, using cell phones, etc.

Visual adverse effects are one of the most relevant causes of visual dissatisfaction, sometimes requiring IOL explantation. De Vries [14] and Woodward [15] investigated the most frequent and relevant causes of low satisfaction among patients undergoing MFIOLs implantation. The most common symptoms are the blurred vision in 94.7% of the eyes implanted with MFIOLs and photic phenomena in circa 40%.

As suggested by Alfonso et al. in [25], the use of aspherical lenses made it possible to compensate for the cornea's spherical aberration and thus reduce the frequency of visual discomfort. In fact, with the use of aspherical refractive MFIOL, Auffarth [16] reported a reduction in the incidence of adverse photic phenomena, with only 3% of patients spontaneously reporting such symptoms.

Our study reported a higher cumulative frequency of adverse photic phenomena (∼60%); however, as discussed above, patients reported that such disturbances occurred rarely, and they did not considerably affect their visual satisfaction. A possible hypothesis that would explain this gap lies in the questionnaire-conducted survey. It is, in fact, conceivable that our patients, despite the explanation provided to them, may have misinterpreted the meaning or description of adverse phenomena. This could have led to an overestimation of the incidence of these symptoms. As further confirmation of this, we recall that patients nevertheless expressed a high degree of satisfaction at all follow-up intervals.

The literature shows that other MFIOL models seem to be associated with higher rates of adverse visual effects, being higher in diffractive MFIOLs. Nuzzi and Tridico [4] found that patients that were implanted with Alcon diffractive SN6AD1 reported a mean glare occurrence rate of 1.20, on a scale from 0 to 5. Cochener et al. [24], testing the FineVision trifocal MFIOL, reported a higher percentage of glare and halos (49% and 31%). Kim et al. [26], who tested the MFIOL TECNIS ZMB00, reported a high incidence of adverse photic effects (49%), resulting in mediocre patients' satisfaction (3/5: neither satisfied nor dissatisfied) despite the good outcomes related to visual acuity. Similarly, Kretz et al. [27] also reported a high percentage of adverse visual phenomena (63%) with overall visual satisfaction of 7.9/10.

A critical parameter in the evaluation of the visual outcomes of MFIOLs is night vision [28]. Aliò et al. [8] significantly correlate night vision with patient satisfaction after surgery, especially while driving. In this sense, the Mplus lens exhibited excellent performance: the average frequency of this disturbance was, in fact, 0.30/5.

## 5. Conclusions

In accordance with the literature, this study confirmed that the Oculentis LENTIS Mplus MF30 is a valid therapeutic alternative for visual rehabilitation after bilateral cataract phacoemulsification surgery in individuals who also want to obtain correction of presbyopia.

MFIOL Oculentis Mplus implantation determined a statistically significant increase in both UCVA and BCVA at 4 m, 60 cm, and 33 cm. We observed a statistically significant improvement in the mean binocular UCVA in the interval t0–t3 at all distances, and we found that VA was already optimal at t3, and no additional lens was needed to further improve VA. Over the subsequent time intervals, the mean binocular UCVA remained constant without statistically significant changes. This supports the already mentioned fact that MFIOLs perform better when bilaterally implanted.

According to our study, the Mplus lens recorded a low average frequency of photic phenomena such as glare and halos and a low frequency of blurred vision both at near and far visual distances. Compared to the data available in the literature, the Mplus lens, because of its design, seems to be effectively associated with a lower frequency of such disturbances.

A remarkable result observed in this study was the finding that six months after surgery the Mplus lens enabled our patients to achieve almost complete independence from the use of spectacles during daily activities. This finding confirms the Mplus MF30's effectiveness in correcting both cataracts and presbyopia.

In conclusion, even if our results leave room for further research, they show the great importance of studying and considering the different characteristics of the numerous IOLs models on the market to achieve maximum patient satisfaction. It seems advisable to put much attention and prudence into considering patients' requests and needs when choosing the right MFIOL since there are differences in their performance and spectacles independence in the execution of daily activities at different visual distances. The increasing knowledge of the technical and clinical characteristics of IOLs and their different use in the replacement of the native lens can lead to the development of more personalized and refraction-oriented cataract surgery, making cataract surgery an exciting opportunity to perform a refractive surgery capable of satisfying even the most demanding requests of patients in terms of independence from the use of spectacles.

## 6. Take-Home Messages

  MFIOLs are a good therapeutic alternative for patients wishing to treat both cataracts and presbyopia.
  Oculentis Mplus MF30 is effective at improving VA at all visual distances at the cost of few visual adverse effects, and it performs equally or better than other premium MFIOLs.
  MFIOL implantation should not be considered as actual refractive surgery but as a way to optimize intraocular refraction and reduce spectacle dependence.

## Figures and Tables

**Table 1:** Monocular 4 m t0-UCVA versus t5-UCVA and t0-BCVA versus t5-BCVA.

4 m					
LogMAR	t0 (preoperative)	t5 (6 months)	Difference	IC 95%	*p* value
UCVA OD	0.57 (±0.23)	0.12 (±0.16)	0.45 (±0.28)	(0.32–0.58)	<0.001
BCVA OD	0.35 (±0.18)	0.07 (±0.15)	0.27 (±0.18)	(0.19–0.36)	<0.001
UCVA OS	0.56 (±0.29)	0.11 (±0.13)	0.45 (±0.32)	(0.30–0.60)	0.001
BCVA OS	0.31 (±0.21)	0.07 (±0.12)	0.24 (±0.22)	(0.14–0.34)	0.006

**Table 2:** Monocular 60 cm t0-UCVA versus t5-UCVA and t0-BCVA versus t5-BCVA.

60 cm					
Jaeger	t0 (preoperative)	t5 (6 months)	Difference	IC 95%	*p* value
UCVA OD	11.1 (±6.0)	2.6 (±1.9)	8.5 (±1.3)	(5.75–11.2)	<0.001
BCVA OD	7.1 (±5.5)	2.0 (±1.7)	5.0 (±1.2)	(2.4–7.6)	0.001
UCVA OS	10.5 (±6.1)	2.3 (±1.7)	8.2 (±1.2)	(5.5–10.8)	<0.001
BCVA OS	6.8 (±6.3)	2.0 (±1.7)	4.8 (±1.3)	(2.0–7.5)	0.002

**Table 3:** Monocular 30 cm t0-UCVA versus t5-UCVA and t0-BCVA versus t5-BCVA.

30 cm					
Jaeger	t0 (preoperative)	t5 (6 months)	Difference	IC 95%	*p* value
UCVA OD	8.6 (±6.4)	1.1 (±0.49)	7.4 (±1.4)	(4.4–10.4)	<0.001
BCVA OD	2.7 (±3.3)	1.0 (±0.2)	1.7 (±0.7)	(0.1–3.2)	0.035
UCVA OS	8.1 (±5.9)	1.2 (±0.6)	6.8 (±1.3)	(4.0–9.6)	<0.001
BCVA OS	3.0 (±3.5)	1.0 (±0.2)	1.9 (±0.8)	(0.2–3.6)	0.026

**Table 4:** Mean binocular 4 m UCVA difference at t0–t3, t3–t4, and t4–t5.

Binocular UCVA 4 m (LogMAR)	t0–t3	t3–t4	t4–t5
Mean difference	0.42 (±0.24)	0.02 (±0.07)	0.01(±0.03)
IC 95%	(0.30–0.53)	(−0.012–0.05)	(−0.004–0.02)
*p*-value	<0.001	0.214	0.163

**Table 5:** Mean binocular 60 cm UCVA difference at t0–t3, t3–t4, and t4–t5.

Binocular UCVA 60 cm (Jaeger)	t0–t3	t3–t4	t4–t5
Mean difference	8.1 (±5.44)	0.00 (±0.32)	0.00 (±0.56)
IC 95%	(5.55–10.64)	(−0.15–0.15)	(−0.26–0.26)
*p*-value	<0.001	1	1

**Table 6:** Mean binocular 60 cm UCVA difference at t0–t3, t3–t4, and t4–t5.

Binocular UCVA 33 cm (Jaejer)	t0–t3	t3–t4	t4–t5
Mean difference	5.4 (±5.4)	0	0.05 (±0.22)
IC 95%	(2.87–7.92)	—	(−0.054–0.15)
*p*-value	<0.001	—	0.33

**Table 7:** Visual satisfaction and spectacle independence.

Q1: satisfaction and spectacle independence	t3	t4	t5
Visual satisfaction	9.20	9.20	9.10
Spectacle independence for far vision	10.00	10.00	10.00
Spectacle independence for near vision	9.50	9.50	9.50
Spectacle independence for intermediate vision	9.60	9.60	9.60

**Table 8:** Visual adverse effects frequency during different follow-up intervals.

Q2: adverse visual effects	t3	t4	t5
Glare	1.20	1.00	1.20
Difficult night vision	0.30	0.30	0.30
Color perception	0.00	0.00	0.00
Depth perception	0.00	0.00	0.00
Halos	0.60	0.80	0.80
Distorted close-up vision	0.00	0.00	0.00
Distorted far vision	0.00	0.20	0.20
Blurred close-up vision	0.40	0.50	0.40
Blurred far vision	0.60	0.40	0.40
Double vision	0.20	0.20	0.10

**Table 9:** Patients' difficulties in carrying out different daily activities.

Q3: daily activities	t3	t4	t5
Watching TV or movies	0.10	0.20	0.10
Playing/working outside	0.00	0.00	0.00
Taking care/playing with children	0.00	0.00	0.00
Read the time on an alarm clock	0.20	0.20	0.20
Read the time on a wall clock	0.50	0.60	0.50
Clear vision at wake-up	0.10	0.10	0.10
Having a hobby	0.20	0.20	020
Recreative activities/sport	0.10	0.10	0.10
Social gatherings	0.10	0.00	0.00
Reading texts/close-up tasks	0.30	0.60	0.50
Cooking *o* doing the shopping	0.20	0.20	0.20
Driving at night	0.40	0.40	0.40
Driving in raining conditions	0.20	0.20	0.20
Using a computer	0.20	0.20	0.20
Using a cellphone	0.10	0.10	0.10
Shaving or putting make up on	0.20	0.20	0.40

## Data Availability

The data generated and analyzed during this study are stored in the repository of the Ophthalmology Department at the A.O.U. Città della Salute *e* della Scienza di Torino, and they are available from the corresponding author on reasonable request.
